# Supplementary material for: Novel Synthesis Methods of New Imidazole-Containing Coordination Compounds Tc(IV, V, VII)—Reaction Mechanism, Xrd and Hirshfeld Surface Analysis
Source: Int J Mol Sci. 2022 Aug 21;23(16):9461. doi: 10.3390/ijms23169461 (PMC9408894; doi:10.3390/ijms23169461)
Supplement: Supplementary file 1 [file ijms-23-09461-s001.zip › ijms-1868144-supplementary.pdf]

Supporting Information

# Novel synthesis methods of new imidazole-containing coordination compounds and salts Tc(IV, V, VII) – reaction mechanism, XRD and Hirshfeld surface analysis

**Mikhail Alexandrovich Volkov *, Anton Petrovich Novikov, Mikhail Semenovich Grigoriev, Alexander Mikhailovich Fedoseev and Konstantin Eduardovich German**

Frumkin Institute of Physical Chemistry and Electrochemistry, Russian Academy of Sciences, Leninskii prospect 31-4, 119071 Moscow, Russia; tony.novickoff@yandex.ru (A.P.N.); mickgrig@mail.ru (M.S.G.); a.fedosseev@gmail.com (A.M.F.); guerman_k@mail.ru (K.E.G.)
* Correspondence: mikhailalexvol@gmail.com

**Table S1.** Bond Lengths for **II** at 100 K**.**

| Atom | Atom | Length/Å | | Atom | Atom | Length/Å |
|---|---|---|---|---|---|---|
| Tc1 | O1 | 1.745(2) | | N4 | C6 | 1.363(5) |
| Tc1 | O2 | 1.755(2) | | N5 | C7 | 1.327(4) |
| Tc1 | N1 | 2.125(3) | | N5 | C8 | 1.372(5) |
| Tc1 | N3 | 2.135(3) | | N6 | C7 | 1.349(4) |
| Tc1 | N5 | 2.122(2) | | N6 | C9 | 1.376(5) |
| Tc1 | N7 | 2.140(3) | | N7 | C10 | 1.322(5) |
| N1 | C1 | 1.323(4) | | N7 | C12 | 1.378(4) |
| N1 | C3 | 1.380(5) | | N8 | C10 | 1.346(5) |
| N2 | C1 | 1.338(5) | | N8 | C11 | 1.374(5) |
| N2 | C2 | 1.360(5) | | C2 | C3 | 1.356(5) |
| N3 | C4 | 1.322(4) | | C5 | C6 | 1.361(5) |
| N3 | C5 | 1.372(5) | | C8 | C9 | 1.358(5) |
| N4 | C4 | 1.335(5) | | C11 | C12 | 1.357(5) |

**Table S2.** Bond Angles for **II** at 100K.

| Atom | Atom | Atom | Angle/° | | Atom | Atom | Atom | Angle/° |
|---|---|---|---|---|---|---|---|---|
| O1 | Tc1 | O2 | 178.40(13) | | C4 | N4 | C6 | 108.5(3) |
| O1 | Tc1 | N1 | 89.24(11) | | C7 | N5 | Tc1 | 124.8(2) |
| O1 | Tc1 | N3 | 88.58(11) | | C7 | N5 | C8 | 106.8(3) |
| O1 | Tc1 | N5 | 89.12(10) | | C8 | N5 | Tc1 | 128.3(2) |
| O1 | Tc1 | N7 | 89.56(10) | | C7 | N6 | C9 | 107.8(3) |
| O2 | Tc1 | N1 | 92.36(12) | | C10 | N7 | Tc1 | 124.4(2) |
| O2 | Tc1 | N3 | 89.82(12) | | C10 | N7 | C12 | 105.9(3) |
| O2 | Tc1 | N5 | 90.78(10) | | C12 | N7 | Tc1 | 129.7(2) |

| | | | | | | | | |
|---|---|---|---|---|---|---|---|---|
| O2 | Tc1 | N7 | 90.56(11) | | C10 | N8 | C11 | 107.8(3) |
| N1 | Tc1 | N3 | 177.79(12) | | N1 | C1 | N2 | 110.5(3) |
| N1 | Tc1 | N7 | 89.67(11) | | C3 | C2 | N2 | 106.7(4) |
| N3 | Tc1 | N7 | 89.95(10) | | C2 | C3 | N1 | 108.6(3) |
| N5 | Tc1 | N1 | 89.66(11) | | N3 | C4 | N4 | 110.5(3) |
| N5 | Tc1 | N3 | 90.67(11) | | C6 | C5 | N3 | 109.6(3) |
| N5 | Tc1 | N7 | 178.53(11) | | C5 | C6 | N4 | 105.4(4) |
| C1 | N1 | Tc1 | 125.3(3) | | N5 | C7 | N6 | 110.0(3) |
| C1 | N1 | C3 | 106.3(3) | | C9 | C8 | N5 | 109.2(3) |
| C3 | N1 | Tc1 | 128.4(2) | | C8 | C9 | N6 | 106.2(3) |
| C1 | N2 | C2 | 108.0(3) | | N7 | C10 | N8 | 110.7(3) |
| C4 | N3 | Tc1 | 127.6(3) | | C12 | C11 | N8 | 105.8(3) |
| C4 | N3 | C5 | 105.9(3) | | C11 | C12 | N7 | 109.7(3) |
| C5 | N3 | Tc1 | 126.6(2) | | | | | |

**Table S3.** Hydrogen Bonds for **II** at 100K.

| D | H | A | d(D-H)/Å | d(H-A)/Å | d(D-A)/Å | D-H-A/° |
|---|---|---|---|---|---|---|
| O1W | H1WA | O2W[1] | 0.85(2) | 1.87(2) | 2.724(4) | 173(4) |
| O1W | H1WB | Br1[1] | 0.86(2) | 2.51(2) | 3.363(3) | 176(5) |
| O2W | H2WA | O1 | 0.83(2) | 1.93(2) | 2.698(3) | 153(4) |
| O2W | H2WB | O2[2] | 0.83(2) | 1.91(2) | 2.694(4) | 156(4) |
| N2 | H2 | O1W[3] | 0.88 | 1.93 | 2.797(4) | 166.7 |
| N4 | H4 | Br1[4] | 0.88 | 2.50 | 3.293(3) | 150.3 |
| N6 | H6 | O2W[1] | 0.88 | 1.93 | 2.783(4) | 162.4 |
| N8 | H8 | Br1 | 0.88 | 2.55 | 3.346(3) | 150.7 |
| C9 | H9 | O1W[5] | 0.95 | 2.41 | 3.108(4) | 129.9 |

[1]+X,1-Y,1/2+Z; [2]1/2+X,1/2+Y,+Z; [3]-1/2+X,3/2-Y,-1/2+Z; [4]+X,-Y,1/2+Z; [5]-1/2+X,-1/2+Y,+Z

**Table S4.** Torsion Angles for **II** at 100 K.

| A | B | C | D | Angle/° | | A | B | C | D | Angle/° |
|---|---|---|---|---|---|---|---|---|---|---|
| Tc1 | N1 | C1 | N2 | 178.0(2) | | C2 | N2 | C1 | N1 | 0.0(4) |
| Tc1 | N1 | C3 | C2 | -177.9(2) | | C3 | N1 | C1 | N2 | -0.2(4) |
| Tc1 | N3 | C4 | N4 | 178.5(2) | | C4 | N3 | C5 | C6 | 0.3(4) |
| Tc1 | N3 | C5 | C6 | -178.1(2) | | C4 | N4 | C6 | C5 | 0.5(4) |
| Tc1 | N5 | C7 | N6 | -179.6(2) | | C5 | N3 | C4 | N4 | 0.0(4) |
| Tc1 | N5 | C8 | C9 | 179.8(2) | | C6 | N4 | C4 | N3 | -0.4(4) |
| Tc1 | N7 | C10 | N8 | 178.8(2) | | C7 | N5 | C8 | C9 | 0.3(4) |
| Tc1 | N7 | C12 | C11 | -178.6(3) | | C7 | N6 | C9 | C8 | 0.2(4) |

| | | | | | | | | | | |
|---|---|---|---|---|---|---|---|---|---|---|
| N2 | C2 | C3 | N1 | -0.3(4) | | C8 | N5 | C7 | N6 | -0.1(4) |
| N3 | C5 | C6 | N4 | -0.5(4) | | C9 | N6 | C7 | N5 | -0.1(4) |
| N5 | C8 | C9 | N6 | -0.3(4) | | C10 | N7 | C12 | C11 | 0.8(4) |
| N8 | C11 | C12 | N7 | -0.6(4) | | C10 | N8 | C11 | C12 | 0.3(4) |
| C1 | N1 | C3 | C2 | 0.3(4) | | C11 | N8 | C10 | N7 | 0.2(5) |
| C1 | N2 | C2 | C3 | 0.2(4) | | C12 | N7 | C10 | N8 | -0.6(4) |

**Table S5.** Bond Lengths for **III** at 100 K.

| Atom | Atom | Length/Å | | Atom | Atom | Length/Å |
|---|---|---|---|---|---|---|
| Tc1 | O1 | 1.763(2) | | N2 | C2 | 1.377(4) |
| Tc1 | O2 | 1.743(2) | | N2 | C1 | 1.343(4) |
| Tc1 | N1 | 2.140(3) | | N2 | C13 | 1.469(4) |
| Tc1 | N3 | 2.130(2) | | N1 | C3 | 1.378(4) |
| Tc1 | N7 | 2.132(3) | | N1 | C1 | 1.331(4) |
| Tc1 | N5 | 2.135(2) | | N3 | C4 | 1.329(4) |
| N6 | C15 | 1.466(4) | | N3 | C5 | 1.376(4) |
| N6 | C9 | 1.374(4) | | N7 | C12 | 1.385(4) |
| N6 | C7 | 1.352(4) | | N7 | C10 | 1.333(4) |
| N8 | C11 | 1.375(4) | | N5 | C8 | 1.377(4) |
| N8 | C16 | 1.462(4) | | N5 | C7 | 1.325(4) |
| N8 | C10 | 1.346(4) | | C3 | C2 | 1.356(4) |
| N4 | C4 | 1.345(4) | | C8 | C9 | 1.363(4) |
| N4 | C6 | 1.372(4) | | C6 | C5 | 1.358(4) |
| N4 | C14 | 1.471(4) | | C11 | C12 | 1.359(4) |

**Table S6.** Bond Angles for **III** at 100 K.

| Atom | Atom | Atom | Angle/° | | Atom | Atom | Atom | Angle/° |
|---|---|---|---|---|---|---|---|---|
| O1 | Tc1 | N1 | 89.58(10) | | C1 | N2 | C13 | 126.1(3) |
| O1 | Tc1 | N3 | 90.56(10) | | C3 | N1 | Tc1 | 124.7(2) |
| O1 | Tc1 | N7 | 89.85(10) | | C1 | N1 | Tc1 | 129.1(2) |
| O1 | Tc1 | N5 | 90.04(10) | | C1 | N1 | C3 | 106.0(3) |
| O2 | Tc1 | O1 | 178.13(10) | | C4 | N3 | Tc1 | 128.7(2) |
| O2 | Tc1 | N1 | 89.78(10) | | C4 | N3 | C5 | 106.1(3) |
| O2 | Tc1 | N3 | 90.07(10) | | C5 | N3 | Tc1 | 125.1(2) |
| O2 | Tc1 | N7 | 88.38(10) | | C12 | N7 | Tc1 | 127.8(2) |
| O2 | Tc1 | N5 | 91.73(10) | | C10 | N7 | Tc1 | 126.2(2) |
| N3 | Tc1 | N1 | 179.75(10) | | C10 | N7 | C12 | 106.0(3) |
| N3 | Tc1 | N7 | 90.41(9) | | C8 | N5 | Tc1 | 126.9(2) |
| N3 | Tc1 | N5 | 89.12(9) | | C7 | N5 | Tc1 | 126.0(2) |
| N7 | Tc1 | N1 | 89.38(9) | | C7 | N5 | C8 | 107.0(3) |
| N7 | Tc1 | N5 | 179.52(9) | | N3 | C4 | N4 | 110.8(3) |

| | | | | | | | | |
|---|---|---|---|---|---|---|---|---|
| N5 | Tc1 | N1 | 91.08(9) | | C2 | C3 | N1 | 109.6(3) |
| C9 | N6 | C15 | 127.4(3) | | C9 | C8 | N5 | 108.6(3) |
| C7 | N6 | C15 | 124.9(3) | | C3 | C2 | N2 | 106.0(3) |
| C7 | N6 | C9 | 107.6(3) | | N1 | C1 | N2 | 110.5(3) |
| C11 | N8 | C16 | 126.9(3) | | C8 | C9 | N6 | 106.7(3) |
| C10 | N8 | C11 | 107.6(3) | | C5 | C6 | N4 | 106.4(3) |
| C10 | N8 | C16 | 125.4(3) | | N5 | C7 | N6 | 110.2(3) |
| C4 | N4 | C6 | 107.5(3) | | C12 | C11 | N8 | 106.6(3) |
| C4 | N4 | C14 | 126.4(3) | | C11 | C12 | N7 | 109.1(3) |
| C6 | N4 | C14 | 126.1(3) | | C6 | C5 | N3 | 109.2(3) |
| C2 | N2 | C13 | 126.0(3) | | N7 | C10 | N8 | 110.7(3) |
| C1 | N2 | C2 | 108.0(3) | | | | | |

**Table S7.** Hydrogen Bonds for **III** at 100 K.

| D | H | A | d(D-H)/Å | d(H-A)/Å | d(D-A)/Å | D-H-A/° |
|---|---|---|---|---|---|---|
| O4 | H4A | O3[1] | 0.85 | 1.94 | 2.775(4) | 167.8 |
| O4 | H4B | Cl1[2] | 0.85 | 2.33 | 3.174(3) | 170.2 |
| O3 | H3A | O1 | 0.85 | 1.93 | 2.778(3) | 177.9 |
| O3 | H3B | Cl1 | 0.85 | 2.52 | 3.296(3) | 151.7 |

[1]+X,1+Y,+Z; [2]1+X,1+Y,+Z

**Table S8.** Torsion Angles for **III** at 100 K.

| A | B | C | D | Angle/° | | A | B | C | D | Angle/° |
|---|---|---|---|---|---|---|---|---|---|---|
| Tc1 | N1 | C3 | C2 | 176.2(2) | | C2 | N2 | C1 | N1 | -0.4(4) |
| Tc1 | N1 | C1 | N2 | -175.88(19) | | C1 | N2 | C2 | C3 | 0.1(4) |
| Tc1 | N3 | C4 | N4 | 174.5(2) | | C1 | N1 | C3 | C2 | -0.5(3) |
| Tc1 | N3 | C5 | C6 | -174.9(2) | | C9 | N6 | C7 | N5 | -0.4(3) |
| Tc1 | N7 | C12 | C11 | 177.9(2) | | C6 | N4 | C4 | N3 | 0.6(4) |
| Tc1 | N7 | C10 | N8 | -177.71(19) | | C7 | N6 | C9 | C8 | 0.4(3) |
| Tc1 | N5 | C8 | C9 | -178.8(2) | | C7 | N5 | C8 | C9 | 0.0(3) |
| Tc1 | N5 | C7 | N6 | 179.00(19) | | C11 | N8 | C10 | N7 | -0.2(3) |
| N8 | C11 | C12 | N7 | -0.4(3) | | C12 | N7 | C10 | N8 | 0.0(3) |
| N4 | C6 | C5 | N3 | -0.3(4) | | C16 | N8 | C11 | C12 | -176.8(3) |
| N1 | C3 | C2 | N2 | 0.2(4) | | C16 | N8 | C10 | N7 | 177.0(3) |
| N5 | C8 | C9 | N6 | -0.2(3) | | C5 | N3 | C4 | N4 | -0.8(4) |
| C15 | N6 | C9 | C8 | 177.9(3) | | C10 | N8 | C11 | C12 | 0.4(3) |
| C15 | N6 | C7 | N5 | -178.0(3) | | C10 | N7 | C12 | C11 | 0.3(3) |
| C4 | N4 | C6 | C5 | -0.2(4) | | C13 | N2 | C2 | C3 | -179.6(3) |
| C4 | N3 | C5 | C6 | 0.7(4) | | C13 | N2 | C1 | N1 | 179.3(3) |

| | | | | | | | | | | |
|---|---|---|---|---|---|---|---|---|---|---|
| C3 | N1 | C1 | N2 | 0.6(3) | | C14 | N4 | C4 | N3 | -178.6(3) |
| C8 | N5 | C7 | N6 | 0.3(3) | | C14 | N4 | C6 | C5 | 179.0(3) |

**Table S9.** Bond Lengths for **IV** at 100 K.

| Atom | Atom | Length/Å | | Atom | Atom | Length/Å |
|---|---|---|---|---|---|---|
| Tc1 | O1[1] | 1.7493(19) | | N1 | C3 | 1.376(4) |
| Tc1 | O1 | 1.7493(19) | | N2 | C1 | 1.347(3) |
| Tc1 | N1[1] | 2.132(2) | | N2 | C2 | 1.369(4) |
| Tc1 | N1 | 2.132(2) | | N2 | C7 | 1.458(3) |
| Tc1 | N3[1] | 2.139(2) | | N3 | C4 | 1.319(4) |
| Tc1 | N3 | 2.139(2) | | N3 | C5 | 1.368(4) |
| Tc2 | O2[2] | 1.710(2) | | N4 | C4 | 1.345(4) |
| Tc2 | O2 | 1.709(2) | | N4 | C6 | 1.364(5) |
| Tc2 | O3 | 1.707(2) | | N4 | C8 | 1.474(4) |
| Tc2 | O3[2] | 1.707(2) | | C2 | C3 | 1.358(4) |
| N1 | C1 | 1.324(3) | | C6 | C5 | 1.349(4) |

[1]1-X,1-Y,1-Z; [2]1-X,+Y,3/2-Z

**Table S10**. Bond Angles for **IV** at 100 K.

| Atom | Atom | Atom | Angle/° | | Atom | Atom | Atom | Angle/° |
|---|---|---|---|---|---|---|---|---|
| O1[1] | Tc1 | O1 | 180.00(9) | | O3 | Tc2 | O3[2] | 110.42(16) |
| O1 | Tc1 | N1[1] | 90.36(9) | | C1 | N1 | Tc1 | 126.12(18) |
| O1[1] | Tc1 | N1[1] | 89.64(9) | | C1 | N1 | C3 | 106.3(2) |
| O1 | Tc1 | N1 | 89.64(9) | | C3 | N1 | Tc1 | 127.44(18) |
| O1[1] | Tc1 | N1 | 90.36(9) | | C1 | N2 | C2 | 107.3(2) |
| O1 | Tc1 | N3 | 89.19(9) | | C1 | N2 | C7 | 125.2(3) |
| O1[1] | Tc1 | N3 | 90.81(9) | | C2 | N2 | C7 | 127.5(3) |
| O1 | Tc1 | N3[1] | 90.81(9) | | C4 | N3 | Tc1 | 128.1(2) |
| O1[1] | Tc1 | N3[1] | 89.19(9) | | C4 | N3 | C5 | 105.9(2) |
| N1 | Tc1 | N1[1] | 180.0 | | C5 | N3 | Tc1 | 126.0(2) |
| N1 | Tc1 | N3[1] | 91.97(8) | | C4 | N4 | C6 | 107.4(3) |
| N1[1] | Tc1 | N3 | 91.97(8) | | C4 | N4 | C8 | 125.6(3) |
| N1 | Tc1 | N3 | 88.03(8) | | C6 | N4 | C8 | 127.0(3) |
| N1[1] | Tc1 | N3[1] | 88.03(8) | | N1 | C1 | N2 | 110.8(2) |
| N3 | Tc1 | N3[1] | 180.0 | | C3 | C2 | N2 | 106.7(3) |
| O2 | Tc2 | O2[2] | 110.7(2) | | C2 | C3 | N1 | 108.9(3) |
| O3 | Tc2 | O2[2] | 109.16(12) | | N3 | C4 | N4 | 110.7(3) |
| O3 | Tc2 | O2 | 108.68(11) | | C5 | C6 | N4 | 106.3(3) |

| | | | | | | | | |
|---|---|---|---|---|---|---|---|---|
| O3[2] | Tc2 | O2[2] | 108.68(11) | | C6 | C5 | N3 | 109.6(3) |
| O3[2] | Tc2 | O2 | 109.17(12) | | | | | |

[1]1-X,1-Y,1-Z; [2]1-X,+Y,3/2-Z

**Table S11.** Hydrogen Bonds for **IV** at 100 K.

| D | H | A | d(D-H)/Å | d(H-A)/Å | d(D-A)/Å | D-H-A/° |
|---|---|---|---|---|---|---|
| C1 | H1 | O3 | 0.95 | 2.48 | 3.183(4) | 130.6 |
| C2 | H2 | O2[1] | 0.95 | 2.45 | 3.267(4) | 144.3 |
| C3 | H3 | O3[2] | 0.95 | 2.56 | 3.229(4) | 127.9 |
| C7 | H7A | O1[3] | 0.98 | 2.39 | 3.163(4) | 135.0 |
| C7 | H7C | O2[4] | 0.98 | 2.40 | 3.331(4) | 159.5 |
| C6 | H6 | O2[5] | 0.95 | 2.37 | 3.250(4) | 153.6 |

[1]1/2+X,-1/2+Y,+Z; [2]3/2-X,-1/2+Y,3/2-Z; [3]1-X,+Y,3/2-Z; [4]3/2-X,3/2-Y,2-Z; [5]+X,2-Y,-1/2+Z

**Table S12.** Torsion Angles for **IV** at 100 K.

| A | B | C | D | Angle/° | | A | B | C | D | Angle/° |
|---|---|---|---|---|---|---|---|---|---|---|
| Tc1 | N1 | C1 | N2 | -175.79(17) | | C3 | N1 | C1 | N2 | -0.3(3) |
| Tc1 | N1 | C3 | C2 | 175.81(19) | | C7 | N2 | C1 | N1 | 177.9(2) |
| Tc1 | N3 | C4 | N4 | -177.59(19) | | C7 | N2 | C2 | C3 | -177.6(3) |
| Tc1 | N3 | C5 | C6 | 177.9(2) | | C4 | N3 | C5 | C6 | 0.2(3) |
| N2 | C2 | C3 | N1 | -0.4(3) | | C4 | N4 | C6 | C5 | 0.5(4) |
| N4 | C6 | C5 | N3 | -0.4(4) | | C6 | N4 | C4 | N3 | -0.4(4) |
| C1 | N1 | C3 | C2 | 0.4(3) | | C5 | N3 | C4 | N4 | 0.1(3) |
| C1 | N2 | C2 | C3 | 0.2(3) | | C8 | N4 | C4 | N3 | 179.5(3) |
| C2 | N2 | C1 | N1 | 0.1(3) | | C8 | N4 | C6 | C5 | -179.4(3) |

**Table S13.** Bond Lengths for **V** at 100 K.

| Atom | Atom | Length/Å | | Atom | Atom | Length/Å |
|---|---|---|---|---|---|---|
| Tc1 | O1 | 1.7034(10) | | N3 | C4 | 1.3773(18) |
| Tc1 | O2 | 1.7105(11) | | N4 | C1 | 1.3307(15) |
| Tc1 | O3 | 1.7080(10) | | N11 | C12 | 1.3402(18) |
| Tc1 | O4 | 1.7246(9) | | N11 | C15 | 1.3737(17) |
| S1 | C1 | 1.7054(12) | | N11 | C16 | 1.4603(18) |
| N1 | C2 | 1.3381(17) | | N13 | C12 | 1.3225(18) |

| | | | | | | |
|---|---|---|---|---|---|---|
| N1 | C5 | 1.3774(17) | | N13 | C14 | 1.3772(17) |
| N1 | C6 | 1.4647(18) | | C4 | C5 | 1.358(2) |
| N2 | C1 | 1.3350(15) | | C14 | C15 | 1.3554(19) |
| N3 | C2 | 1.3256(16) | | | | |

**Table S14.** Bond Angles for **V** at 100 K.

| Atom | Atom | Atom | Angle/° | | Atom | Atom | Atom | Angle/° |
|---|---|---|---|---|---|---|---|---|
| O1 | Tc1 | O2 | 108.91(6) | | C15 | N11 | C16 | 126.04(12) |
| O1 | Tc1 | O3 | 110.04(6) | | C12 | N13 | C14 | 106.37(12) |
| O1 | Tc1 | O4 | 109.38(5) | | N2 | C1 | S1 | 121.39(9) |
| O2 | Tc1 | O4 | 109.33(5) | | N4 | C1 | S1 | 120.88(9) |
| O3 | Tc1 | O2 | 109.64(6) | | N4 | C1 | N2 | 117.72(11) |
| O3 | Tc1 | O4 | 109.53(5) | | N3 | C2 | N1 | 109.89(11) |
| C2 | N1 | C5 | 108.03(11) | | C5 | C4 | N3 | 108.23(12) |
| C2 | N1 | C6 | 125.32(12) | | C4 | C5 | N1 | 106.51(12) |
| C5 | N1 | C6 | 126.66(12) | | N13 | C12 | N11 | 110.71(12) |
| C2 | N3 | C4 | 107.34(11) | | C15 | C14 | N13 | 108.93(12) |
| C12 | N11 | C15 | 107.57(11) | | C14 | C15 | N11 | 106.42(12) |
| C12 | N11 | C16 | 126.36(12) | | | | | |

**Table S15.** Hydrogen Bonds for **V** at 100 K.

| D | H | A | d(D-H)/Å | d(H-A)/Å | d(D-A)/Å | D-H-A/° |
|---|---|---|---|---|---|---|
| N2 | H2A | O3[1] | 0.88 | 2.39 | 2.8943(15) | 116.8 |
| N2 | H2A | O4[2] | 0.88 | 2.26 | 3.0230(15) | 144.6 |
| N2 | H2B | S1[1] | 0.88 | 2.57 | 3.4477(11) | 171.9 |
| N3 | H3 | N13 | 0.88 | 1.85 | 2.7246(17) | 172.3 |
| N4 | H4A | O1[3] | 0.88 | 2.43 | 2.8781(15) | 112.3 |
| N4 | H4A | O4[2] | 0.88 | 2.16 | 2.9460(14) | 148.5 |
| N4 | H4B | S1[3] | 0.88 | 2.54 | 3.4063(11) | 169.8 |
| N13 | H13 | N3 | 0.88 | 1.86 | 2.7246(17) | 168.7 |
| C2 | H2 | O3 | 0.95 | 2.27 | 3.1585(16) | 156.2 |
| C4 | H4 | S1[4] | 0.95 | 2.92 | 3.8357(14) | 161.1 |
| C5 | H5 | O1[5] | 0.95 | 2.65 | 3.4910(19) | 148.3 |
| C14 | H14 | S1[4] | 0.95 | 2.80 | 3.7200(14) | 164.1 |
| C15 | H15 | O4[6] | 0.95 | 2.53 | 3.4443(17) | 162.6 |
| C16 | H16C | O2[6] | 0.98 | 2.59 | 3.5054(19) | 156.2 |

[1]1/2-X,-1/2+Y,1/2-Z; [2]-1+X,+Y,+Z; [3]1/2-X,1/2+Y,1/2-Z; [4]1-X,1-Y,1-Z; [5]1/2+X,1/2-Y,1/2+Z; [6]-1/2+X,3/2-Y,1/2+Z

**Table S16.** Torsion Angles for **V** at 100 K.

| A | B | C | D | Angle/° | | A | B | C | D | Angle/° |
|---|---|---|---|---|---|---|---|---|---|---|
| N3 | C4 | C5 | N1 | -0.44(16) | | C6 | N1 | C5 | C4 | -179.78(12) |
| N13 | C14 | C15 | N11 | 0.20(16) | | C12 | N11 | C15 | C14 | -0.08(15) |
| C2 | N1 | C5 | C4 | 0.26(15) | | C12 | N13 | C14 | C15 | -0.25(16) |
| C2 | N3 | C4 | C5 | 0.46(15) | | C14 | N13 | C12 | N11 | 0.20(15) |
| C4 | N3 | C2 | N1 | -0.30(15) | | C15 | N11 | C12 | N13 | -0.08(15) |
| C5 | N1 | C2 | N3 | 0.03(15) | | C16 | N11 | C12 | N13 | 178.11(12) |
| C6 | N1 | C2 | N3 | -179.93(12) | | C16 | N11 | C15 | C14 | -178.27(13) |

**Table S17.** Bond Lengths for **VI** at 296 K.

| Atom | Atom | Length/Å | | Atom | Atom | Length/Å |
|---|---|---|---|---|---|---|
| Tc01 | Cl02[1] | 2.3378(12) | | N2 | C3 | 1.3165 |
| Tc01 | Cl02 | 2.3378(12) | | N2 | C6 | 1.3788 |
| Tc01 | Cl03 | 2.3642(11) | | C3 | N4 | 1.3685 |
| Tc01 | Cl03[1] | 2.3641(11) | | N4 | C5 | 1.3816 |
| Tc01 | Cl04[1] | 2.3392(12) | | C5 | C6 | 1.3748 |
| Tc01 | Cl04 | 2.3392(12) | | | | |

[1] 1-X,1-Y,1-Z

**Table S18.** Bond Angles for **VI** at 296 K.

| Atom | Atom | Atom | Angle/° | | Atom | Atom | Atom | Angle/° |
|---|---|---|---|---|---|---|---|---|
| Cl02[1] | Tc01 | Cl02 | 180.0 | | Cl04 | Tc01 | Cl03 | 90.0 |
| Cl02[1] | Tc01 | Cl03[1] | 91.05(5) | | Cl04[1] | Tc01 | Cl03 | 90.0 |
| Cl02[1] | Tc01 | Cl03 | 88.95(5) | | Cl04[1] | Tc01 | Cl03[1] | 90.0 |
| Cl02 | Tc01 | Cl03 | 91.06(5) | | Cl04 | Tc01 | Cl03[1] | 90.0 |
| Cl02 | Tc01 | Cl03[1] | 88.94(5) | | Cl04[1] | Tc01 | Cl04 | 180.0 |
| Cl02[1] | Tc01 | Cl04[1] | 90.0 | | C3 | N2 | C6 | 105.4 |
| Cl02 | Tc01 | Cl04 | 90.0 | | N2 | C3 | N4 | 111.6 |

| | | | | | | | | |
|---|---|---|---|---|---|---|---|---|
| Cl02[1] | Tc01 | Cl04 | 90.0 | | C3 | N4 | C5 | 107.3 |
| Cl02 | Tc01 | Cl04[1] | 90.0 | | C6 | C5 | N4 | 105.0 |
| Cl03[1] | Tc01 | Cl03 | 180.0 | | C5 | C6 | N2 | 110.7 |

[1]1-X,1-Y,1-Z

**Table S19.** Hydrogen Bonds for **VI** at 296 K.

| **D** | **H** | **A** | **d(D-H)/Å** | **d(H-A)/Å** | **d(D-A)/Å** | **D-H-A/°** |
|---|---|---|---|---|---|---|
| N2 | H2 | Cl02[1] | 0.86 | 2.56 | 3.330(9) | 149.2 |
| N4 | H4 | Cl03 | 0.86 | 2.68 | 3.340(5) | 135.0 |
| N4 | H4 | Cl04[2] | 0.86 | 2.83 | 3.351(5) | 120.7 |

[1]3/2-X,1/2+Y,2-Z; [2]1/2+X,-1/2+Y,+Z

**Table S20.** Torsion Angles for **VI** at 296 K.

| **A** | **B** | **C** | **D** | **Angle/°** | | **A** | **B** | **C** | **D** | **Angle/°** |
|---|---|---|---|---|---|---|---|---|---|---|
| N2 | C3 | N4 | C5 | 0.0 | | N4 | C5 | C6 | N2 | 0.0 |
| C3 | N2 | C6 | C5 | 0.0 | | C6 | N2 | C3 | N4 | 0.0 |
| C3 | N4 | C5 | C6 | 0.0 | | | | | | |

**Table S21.** Atomic Occupancy for **VI** at 296 K.

| **Atom** | ***Occupancy*** | | **Atom** | ***Occupancy*** | | **Atom** | ***Occupancy*** |
|---|---|---|---|---|---|---|---|
| N2 | 0.5 | | H2 | 0.5 | | C3 | 0.5 |
| H3 | 0.5 | | N4 | 0.5 | | H4 | 0.5 |
| C5 | 0.5 | | H5 | 0.5 | | C6 | 0.5 |
| H6 | 0.5 | | | | | | |

**Table S22.** Bond Lengths for **VII** at 296 K.

| **Atom** | **Atom** | **Length/Å** | | **Atom** | **Atom** | **Length/Å** |
|---|---|---|---|---|---|---|
| Tc1 | Br1 | 2.5541(6) | | N2 | C1 | 1.285(8) |
| Tc1 | Br1[1] | 2.5540(6) | | N2 | C3 | 1.349(8) |
| Tc1 | Br2 | 2.5218(6) | | N5 | C1 | 1.307(9) |
| Tc1 | Br2[1] | 2.5218(6) | | N5 | C4 | 1.376(9) |
| Tc1 | Br3[1] | 2.5227(6) | | C3 | C4 | 1.297(9) |

| | | | | | | |
|---|---|---|---|---|---|---|
| Tc1 | Br3 | 2.5227(6) | | | | |

[1]1-X,1-Y,1-Z

**Table S23.** Bond Angles for **VII** at 296 K.

| Atom | Atom | Atom | Angle/° | | Atom | Atom | Atom | Angle/° |
|---|---|---|---|---|---|---|---|---|
| Br1[1] | Tc1 | Br1 | 180.0 | | Br3 | Tc1 | Br1 | 90.951(19) |
| Br2 | Tc1 | Br1[1] | 90.545(19) | | Br3 | Tc1 | Br1[1] | 89.05(2) |
| Br2[1] | Tc1 | Br1[1] | 89.454(19) | | Br3[1] | Tc1 | Br1 | 89.05(2) |
| Br2[1] | Tc1 | Br1 | 90.546(19) | | Br3[1] | Tc1 | Br1[1] | 90.95(2) |
| Br2 | Tc1 | Br1 | 89.455(19) | | Br3[1] | Tc1 | Br3 | 180.0 |
| Br2 | Tc1 | Br2[1] | 180.0 | | C1 | N2 | C3 | 109.5(6) |
| Br2 | Tc1 | Br3[1] | 89.872(16) | | C1 | N5 | C4 | 109.5(5) |
| Br2 | Tc1 | Br3 | 90.127(16) | | N2 | C1 | N5 | 107.2(6) |
| Br2[1] | Tc1 | Br3 | 89.873(16) | | C4 | C3 | N2 | 108.6(6) |
| Br2[1] | Tc1 | Br3[1] | 90.128(16) | | C3 | C4 | N5 | 105.1(6) |

[1]1-X,1-Y,1-Z

**Table S24.** Hydrogen Bonds for **VII** at 296 K.

| D | H | A | d(D-H)/Å | d(H-A)/Å | d(D-A)/Å | D-H-A/° |
|---|---|---|---|---|---|---|
| N2 | H2 | Br2[1] | 0.86 | 2.82 | 3.500(5) | 136.8 |
| N2 | H2 | Br3[2] | 0.86 | 2.91 | 3.582(6) | 136.5 |
| N5 | H5 | Br1 | 0.86 | 2.77 | 3.488(5) | 141.6 |

[1]-1+X,3/2-Y,-1/2+Z; [2]-X,1/2+Y,1/2-Z

**Table S25.** Torsion Angles for **VII** at 296 K.

| A | B | C | D | Angle/° | | A | B | C | D | Angle/° |
|---|---|---|---|---|---|---|---|---|---|---|
| N2 | C3 | C4 | N5 | 0.8(7) | | C3 | N2 | C1 | N5 | -1.6(7) |
| C1 | N2 | C3 | C4 | 0.5(7) | | C4 | N5 | C1 | N2 | 2.1(8) |
| C1 | N5 | C4 | C3 | -1.8(8) | | | | | | |

**Table S26.** Bond Lengths for **VIII** at 100 K.

| Atom | Atom | Length/Å | | Atom | Atom | Length/Å |
|---|---|---|---|---|---|---|
| Sn1 | Cl2 | 2.3987(4) | | N4 | C17 | 1.331(2) |
| Sn1 | Cl3 | 2.4099(4) | | N4 | C19 | 1.372(2) |
| Sn1 | Cl4 | 2.4079(4) | | N5 | C14 | 1.325(2) |
| Sn1 | Cl5 | 2.4221(4) | | N5 | C15 | 1.376(2) |
| Sn1 | Cl7 | 2.3942(4) | | N5 | C24 | 1.464(2) |
| Sn1 | Cl9 | 2.4103(4) | | C7 | C11 | 1.347(2) |
| N1 | C6 | 1.327(2) | | N8 | C13 | 1.325(2) |
| N1 | C11 | 1.369(2) | | N8 | C23 | 1.370(2) |
| N2 | C6 | 1.326(2) | | N9 | C17 | 1.326(2) |
| N2 | C7 | 1.377(2) | | N9 | C21 | 1.370(2) |
| N2 | C10 | 1.462(2) | | N12 | C14 | 1.317(2) |
| N3 | C13 | 1.3273(19) | | N12 | C18 | 1.367(2) |
| N3 | C20 | 1.373(2) | | C15 | C18 | 1.344(2) |
| N3 | C22 | 1.460(2) | | C19 | C21 | 1.338(2) |
| N4 | C16 | 1.463(2) | | C20 | C23 | 1.343(2) |

**Table S27.** Bond Angles for **VIII** at 100 K.

| Atom | Atom | Atom | Angle/° | | Atom | Atom | Atom | Angle/° |
|---|---|---|---|---|---|---|---|---|
| Cl2 | Sn1 | Cl3 | 90.638(13) | | C17 | N4 | C16 | 125.65(14) |
| Cl2 | Sn1 | Cl4 | 89.599(13) | | C17 | N4 | C19 | 108.39(14) |
| Cl2 | Sn1 | Cl5 | 90.288(13) | | C19 | N4 | C16 | 125.95(14) |
| Cl2 | Sn1 | Cl9 | 179.169(14) | | C14 | N5 | C15 | 108.40(14) |
| Cl3 | Sn1 | Cl5 | 88.943(14) | | C14 | N5 | C24 | 125.95(16) |
| Cl3 | Sn1 | Cl9 | 90.139(14) | | C15 | N5 | C24 | 125.65(15) |
| Cl4 | Sn1 | Cl3 | 90.819(14) | | N2 | C6 | N1 | 108.19(14) |
| Cl4 | Sn1 | Cl5 | 179.736(16) | | C11 | C7 | N2 | 107.01(14) |
| Cl4 | Sn1 | Cl9 | 90.097(13) | | C13 | N8 | C23 | 108.92(14) |
| Cl7 | Sn1 | Cl2 | 89.769(14) | | C17 | N9 | C21 | 108.68(14) |
| Cl7 | Sn1 | Cl3 | 178.146(14) | | C7 | C11 | N1 | 106.96(14) |
| Cl7 | Sn1 | Cl4 | 90.992(15) | | C14 | N12 | C18 | 109.11(15) |
| Cl7 | Sn1 | Cl5 | 89.246(15) | | N8 | C13 | N3 | 108.18(14) |
| Cl7 | Sn1 | Cl9 | 89.463(14) | | N12 | C14 | N5 | 108.54(15) |
| Cl9 | Sn1 | Cl5 | 90.020(13) | | C18 | C15 | N5 | 106.99(14) |
| C6 | N1 | C11 | 109.12(14) | | N9 | C17 | N4 | 108.34(14) |
| C6 | N2 | C7 | 108.71(13) | | C15 | C18 | N12 | 106.97(15) |
| C6 | N2 | C10 | 125.90(14) | | C21 | C19 | N4 | 107.31(14) |
| C7 | N2 | C10 | 125.32(14) | | C23 | C20 | N3 | 107.03(14) |
| C13 | N3 | C20 | 108.74(14) | | C19 | C21 | N9 | 107.28(14) |
| C13 | N3 | C22 | 125.36(14) | | C20 | C23 | N8 | 107.12(15) |
| C20 | N3 | C22 | 125.82(13) | | | | | |

**Table S28.** Hydrogen Bonds for **VIII** at 100 K

| Table 6 Hydrogen Bonds for VIII at 296 K. | | | | | | |
|---|---|---|---|---|---|---|
| **D** | **H** | **A** | **d(D-H)/Å** | **d(H-A)/Å** | **d(D-A)/Å** | **D-H-A/°** |
| N1 | H1 | Cl1 | 0.87(2) | 2.22(2) | 3.0741(15) | 167.4(18) |
| N8 | H8 | Cl6[1] | 0.86(2) | 2.23(2) | 3.0836(15) | 171.2(19) |
| N9 | H9 | Cl1[2] | 0.88(2) | 2.23(2) | 3.0810(15) | 164.1(18) |
| N12 | H12 | Cl6 | 0.82(2) | 2.24(2) | 3.0563(15) | 174(2) |

[1]1-X,1/2+Y,1/2-Z; [2]2-X,2-Y,1-Z

**Table S29.** Torsion Angles for **VIII** at 100 K.

| A | B | C | D | Angle/° | | A | B | C | D | Angle/° |
|---|---|---|---|---|---|---|---|---|---|---|
| N2 | C7 | C11 | N1 | -0.05(17) | | C15 | N5 | C14 | N12 | -0.50(19) |
| N3 | C20 | C23 | N8 | 0.26(19) | | C16 | N4 | C17 | N9 | 178.71(14) |
| N4 | C19 | C21 | N9 | -0.11(18) | | C16 | N4 | C19 | C21 | -178.64(15) |
| N5 | C15 | C18 | N12 | -0.40(18) | | C17 | N4 | C19 | C21 | 0.08(18) |
| C6 | N1 | C11 | C7 | 0.00(18) | | C17 | N9 | C21 | C19 | 0.11(18) |
| C6 | N2 | C7 | C11 | 0.09(17) | | C18 | N12 | C14 | N5 | 0.2(2) |
| C7 | N2 | C6 | N1 | -0.09(17) | | C19 | N4 | C17 | N9 | -0.02(17) |
| C10 | N2 | C6 | N1 | -177.20(14) | | C20 | N3 | C13 | N8 | 0.09(18) |
| C10 | N2 | C7 | C11 | 177.22(14) | | C21 | N9 | C17 | N4 | -0.06(17) |
| C11 | N1 | C6 | N2 | 0.06(18) | | C22 | N3 | C13 | N8 | -176.86(15) |
| C13 | N3 | C20 | C23 | -0.22(19) | | C22 | N3 | C20 | C23 | 176.71(16) |
| C13 | N8 | C23 | C20 | -0.21(19) | | C23 | N8 | C13 | N3 | 0.07(18) |
| C14 | N5 | C15 | C18 | 0.56(19) | | C24 | N5 | C14 | N12 | 179.70(16) |
| C14 | N12 | C18 | C15 | 0.1(2) | | C24 | N5 | C15 | C18 | -179.64(16) |
